# DNA barcode for the identification of the sand fly *Lutzomyia longipalpis* plant feeding preferences in a tropical urban environment

**DOI:** 10.1038/srep29742

**Published:** 2016-07-20

**Authors:** Leonardo H. G. de M. Lima, Marcelo R. Mesquita, Laura Skrip, Moisés T. de Souza Freitas, Vladimir C. Silva, Oscar D. Kirstein, Ibrahim Abassi, Alon Warburg, Valdir de Q. Balbino, Carlos H. N. Costa

**Affiliations:** 1 Federal University of Piauí, Picos, Brazil; 2 Laboratory of Leishmaniasis, Teresina, Brazil; 3 Federal Institute of Piauí, Parnaíba, Brazil; 4 Center for Infectious Disease Modeling and Analysis, Yale School of Public Health, New Haven, Connecticut, United States; 5 Laboratory of Bioinformatics and Evolutionary Biology, Federal University of Pernambuco, Recife, Brazil; 6 Department of Microbiology and Molecul Genetics, The Institute of Medical Research Israel-Canada, The Kuvin Centre for the Study of Infectious and Tropical Diseases, The Hebrew University of Jerusalem, Jerusalem, Israel

## Abstract

Little is known about the feeding behavior of hematophagous insects that require plant sugar to complete their life cycles. We studied plant feeding of *Lutzomyia longipalpis* sand flies, known vectors of *Leishmania infantum/chagasi* parasites, in a Brazilian city endemic with visceral leishmaniasis. The DNA barcode technique was applied to identify plant food source of wild-caught *L. longipalpis* using specific primers for a locus from the chloroplast genome, ribulose diphosphate carboxylase. DNA from all trees or shrubs within a 100-meter radius from the trap were collected to build a barcode reference library. While plants from the Anacardiaceae and Meliaceae families were the most abundant at the sampling site (25.4% and 12.7% of the local plant population, respectively), DNA from these plant families was found in few flies; in contrast, despite its low abundance (2.9%), DNA from the Fabaceae family was detected in 94.7% of the sand flies. The proportion of sand flies testing positive for DNA from a given plant family was not significantly associated with abundance, distance from the trap, or average crown expansion of plants from that family. The data suggest that there may indeed be a feeding preference of *L. longipalpis* for plants in the Fabaceae family.

Visceral leishmaniasis is a lethal vectorborne disease caused by protozoa from the genus *Leishmania*, and in recent decades, it has increasingly affected populations in urban settings of Brazil^1^. The first major urban outbreak took place in Teresina, Piauí State, in the early 1980s after a massive planting of acacias. It has thus been hypothesized that the two events were connected.

The quantity and diversity of flora in a habitat are associated with the quality and availability of resources to insects^2^. Plants supply insects with food, protection against predators and adequate shelter to lay their eggs^3^,^4^. Accordingly, environmental changes related to plant types and abundance can modify the behavior of disease vectors^5^.

Interactions between sand flies, the vectors of *Leishmania* parasites, and vegetation have been considered^6^,^7^. Costa specifically suggested a relation between acacias, which attract sand flies of the species *Lutzomyia longipalpis* and offer protection against predators, and vector proliferation in areas where these plants are abundant^8^. The relationship between acacias and visceral leishmaniasis vectors has been previously considered since one of the largest known epidemics of the disease was observed in Sudan among displaced people living in areas with abundant acacia trees^9^.

Sand flies require sugar as their energy source^10^,^11^,^12^. However, there is little information about specific sources of these sugars and about how sand flies are attracted to plants, especially under natural conditions^7^,^13^.

To extend current knowledge about the ecology of insects and plants, more information about insect-plant interactions is necessary^14^. Direct analysis of the plant content in an insect’s digestive tract is a precise approach to understanding feeding behavior^15^.

The use of the DNA barcode tool, a technique that is able to identify the food content in invertebrates, using short sequences obtained in conserved regions of the chloroplast, has been widely reported in the literature^16^,^17^,^18^,^19^. Specifically, the ribulose diphosphate carboxylase (*rbcL*) gene has been successfully used to identify food source^14^,^20^,^21^. This same locus was used by Junnila *et al*.^22^ to identify plant-derived food content in wild-caught *Phlebotomus papatasi* sand flies.

The DNA barcode approach can be used to identify relationships between presence of plant types and vectorial capacity or simply the likelihood of vector habitats in a particular area. One application of this technique, therefore, would be informing vector control measures, such as urban landscaping techniques, for leishmaniasis and other vectorborne diseases. Here, we describe a study designed to identify plant-based feeding preferences of *L. longipalpis* through the use of DNA barcode technology in a Brazilian city where visceral leishmaniasis is endemic.

## Results

**Figure 1:**
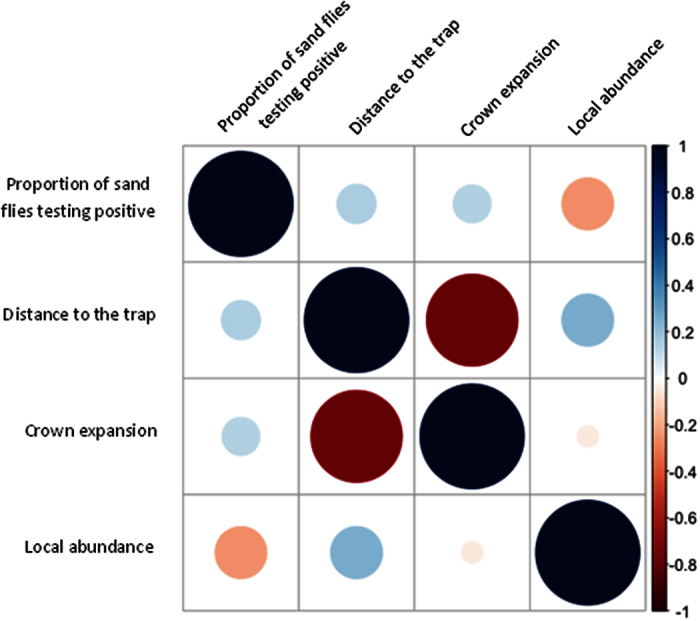
Correlations between the proportion of sand flies testing positive for DNA from a given plant family and plant characteristics. Red represents a negative correlation and blue represents a positive correlation. Larger circles represent stronger correlations. Relationships are presented for (**A**) proportion of sand flies testing positive for DNA from a given plant family, (**B**) average distance of plants from a given family to a trap, (**C**) average crown expansion of plants from a given family, and (**D**) local abundance of plants from a given family. A strong negative correlation was observed between the distance of plants from a given family to the trap and the average crown expansion of plants from that family.

We used *rbcL* PCR to detect and identify plant DNA in the guts of 100 *L. longipalpis* captured during 5 days in the tropical urban environment of Teresina, Piauí State. Fifty-seven percent of the flies were found positive for plant DNA. In the vicinity of the trapping location, we identified 22 species of plants belonging to 14 families (Supplementary Table S1). DNA from the ubiquitous plant family Fabaceae was most prevalent and was identified in 94.7% (54/57) of sand flies in which plant DNA was detected. On average, each sand fly was positive for the DNA of 2.1 ± 1.4 plant families. To consider whether plant availability or proximity may have contributed to observed feeding trends among sand flies, relationships between plant characteristics and frequency of plant DNA detection in sand fly guts were assessed. The proportion of sand flies testing positive for DNA of a given plant family was not significantly correlated with local abundance of plants from that family, average distance of plants from the trap, or average crown expansion of plants in the family (Fig. 1). A statistically significant, negative correlation was found between the average distance between plants from a family and the trap and their average crown expansion (Pearson r = −0.77; p = 0.002) (Fig. 1 and Supplementary Table S1).

On each of the five trap days, DNA from four to eight of the plant families was detected in the flies captured and analyzed (Table 1). Although there was little variation in measurements of abiotic factors (*i.e*., temperature, air velocity, relative humidity) across the sampling period, humidity was highest on the two days with lower temperatures (Day 1 and Day 4). DNA from 7.5 plant families on average was detected in sand flies captured on these two cooler, more humid days, while DNA from 5 plant families on average was detected in sand flies captured on the warmer, less humid days.

## Discussion

This study verified the feasibility of detecting plant DNA in the digestive tracts of *L. longipalpis* using a barcode approach. The successful application of this approach is consistent with previous studies investigating feeding behavior of *P. papatasi*^22^. Furthermore, detection of plant DNA in sand fly guts is biologically plausible as sand flies feed directly from the plant’s tissue^23^,^24^ to obtain sugars that will be used as energy sources^25^.

However, despite the success of the approach presented here in distinguishing *L. longipalpis* feeding sources, plant DNA was not detected in nearly half of the caught sand flies. This may be explained by factors such as DNA degradation by enzymes in the digestive tract—a process that occurs with blood DNA ingested by females during blood feeding^26^,^27^ — or due to sugar acquisition from honeydew excreted by aphids and coccidians^28^,^29^.

At the sand fly trapping site, plant species belonging to 14 families were collected and identified. The Fabaceae family was the most frequently detected food source of *L. longipalpis* (Table 2) despite the low abundance (2.9% of local plants) and relatively high average distance from the trap of plants from this family. Additionally, DNA from plant families, specifically Anacardiacea and Meliaceae, that were most represented in the area (*i.e*., plants from these families had the highest abundance) was found in fewer insects than DNA from the Fabaceae family (Table 2 and Supplementary Table S1). Of further note, some plant families (*i.e*., Rutacea and Annonacea) with abundance higher than that of Fabaceae were found in none of the sand flies (Table 2). This attests to the fact that sand flies have a feeding preference for certain plants or plant families.

The plant families Fabaceae, Anacardiaceae, Meliciaeceae, Rutaceae and Annonacea have pantropical distributions. The Fabaceae family, commonly known as legumes, is considered to be the third largest angiosperm family^30^. The species belonging to this family have carbohydrate heterogeneity, varying from simple sugar to complex heteropolysaccharides^31^. The Anacardiaceae family is known for having many fruit trees of high economic value, due to their wood and their production of substances used in industry or medicine^32^. The Meliaceae family includes trees producing meliacin, known for its insecticidal properties^33^. Plants from the Rutaceae family have a large variety of secondary metabolites, as alkaloids, coumarins, flavonoids, limonoids, and volatile oils^34^. Species belonging to the Annonaceae family produce bio compounds with medicinal, allelopathic or pesticide properties^35^.

The attraction of sand flies to certain plants, as well as to certain honey odors, has been reported in different studies^36^,^37^,^38^,^39^. According to Muller and Schlein^12^ sand flies seeking sugar sources are primarily guided by attractive factors. These factors can be the carbohydrate composition of certain plants or plant families, high CO_2_ emission which can be detected by the insect^40^, or even the release of a phytochemical affecting the olfactory system^41^,^42^.

Muller *et al*.^13^ who evaluated the *P. papatasi* attraction index for different plant species, found that phytochemicals serve as potential attractants for insects. Magalhaes-Junior *et al*.^43^ additionally verified that volatile plant compounds, 1-octen-3-ol, 1-nonanol and 1-heptanol, can act as attractive factors for insects from the *L. longipalpis* species. It is worth noting that the compounds 1-octen-3-ol^44^ and 1-nonanol^45^ have been identified in different species belonging to the Fabaceae family.

Through an assessment of the relationship between abiotic factors and the number of families detected in sand flies per collection day, it was evidenced that more plant families were used as a feeding sources on those days with lower temperatures and higher relative humidity. However, no reports were found in the literature about how these factors might influence the dynamics of sugar acquisition in these insects. Currently, it is known that abiotic factors such as temperature and relative humidity influence the population dynamics in the different sand fly species^46^,^47^,^48^ including *L. longipalpis*^49^.

Accordingly, our study suggested there is indeed a feeding preference by *L. longipalpis* for the Fabaceae family, represented in this study by specimens of the species *Albizia niopoides, Anadenanthera macrocarpa, Cenostigma macrophyllum* and *Tamarindus indica*. At the same time, the data suggests that *L. longipalpis* had less preference for plants belonging to the families Anacardiacea, Meliaceae, Rutacea or Annonacea.

Upon further verification of *L. longipalpis* plant feeding preferences, it will be necessary to determine whether sand fly abundance varies with the presence or absence of plants from certain families. If so, the combined findings may have implications for controlling urban epidemics of vector borne disease. In particular, removal of preferred sugar sources for sand flies could reduce their prevalence and thus reduce density-dependent transmission of the pathogens they harbor.

It is also recommended that new studies relating the feeding interaction between sand flies and plants focus not only in the identification of plant species or plant families serving as a carbohydrate source, but also in the identification of the chemical components that lead to the insect preference for certain food source. Additionally, it is necessary to understand the role of each abiotic factor in this feeding process, and this way, sand fly population control measures may be applied.

## Materials and Methods

### Field collection of phlebotomine sand flies

Insect and plant collection were conducted in a region of the city of Teresina (05°07′700″S/42°46′426″W), capital of Piauí State, in Brazil’s Northeast. The sand flies were captured using a CDC light trap, deployed in an animal shelter outside of a household. The trap remained at the site for five consecutive nights working 12 hours per night (6 P.M. to 6 A.M.) in January, 2015. The captured sand flies were taken to the laboratory for identification and processing. Only *L. longipalpis* sand flies were separated randomly from the sample of sand flies collected every trapping night. In order to avoid the contamination of the samples during the DNA extraction process, every plant fragment was removed from the sand fly bodies. Previously collected insects were submerged in a solution of 0.5% hypochlorite with 0.01 ml/ml Triton X-100 detergent, agitated gently for 1 min with forceps, and then rinsed in double distilled water (ddH20) for 1 min as proposed by Matheson *et al*.^15^. After this procedure, the insects were stored at −80 °C for further DNA extraction. All the techniques used for plant identification had been previously tested using DNA directly from plants (Lima *et al*., submitted). For every capture day, values of temperature, relative humidity and wind speed were obtained in collaboration with the National Meteorology Institute (INMET).

### Field collection of plants

The botanical material (leaves and flowers) was collected under the criteria that plants should be, at the most, 100 meters apart from the sand fly trap, which is within the interval of dispersal of the sand fly *Lu. longipalpis*^50^. In total, 22 plant species were collected and subsequently identified at the Graziela Barroso Herbarium at the Federal University of Piauí. For genetic analysis, species were labeled and stored at −80 °C until DNA extraction. Information on species included in the present study is provided in Supplementary Table S1.

Distances of each plant specimen from the CDC traps were recorded using a Garmin Dakota 10 GPS device (Garmin, USA) and the average expansion of the crown was measured according to methods proposed by Blozan^51^.

### DNA extraction, PCR and sequencing

During the DNA extraction process, sand flies were put in 1.5 mL test tubes and 300 μl of CTAB lysis buffer (50 mL 2x CTAB + 50 mL ddH^2^0 + 200 mL mercaptoethanol (0.2%)) was added. The samples were macerated with an Argos Pellet Mixer (Argos, USA), and 10 μl K proteinase (Invitrogen, USA) was added to each tube (concentration 10 mg/mL). Immediately after, the samples were incubated for two hours in a 60 °C hot bath. Extraction was then conducted using the PureLink Genomic DNA Mini Kit (Invitrogen, USA) according to the manufacturer’s instructions. The plant genomic DNA extraction was carried out using the kit BIOPUR Extração Mini Spin Planta (Biometrix, Brazil). The DNA was extracted and analyzed in 1% agarose gel under UV light and subsequently was quantified by spectrophotometer NanoDrop™ 2000 (ThermoScientific, USA) and the fluorometer Qubit^®^ 2.0 (Invitrogen, USA).

For each separated DNA sample, a segment of chloroplast gene *rbcL* was amplified by PCR using the PCR Master Mix (Promega, USA) according to the manufacturer’s instructions for a 50 μL final volume, containing 1 μL of each primer (rbcLaF and rbcLaR^52^), and 5 μL DNA template. The reactions took place in a T100 (Bio-Rad, USA) thermal cycler. The thermal cycle conditions have been described by Bafeel *et al*.^52^. PCR products were visualized in 1% agarose gel under UV light and purified using ExoSap-IT (Affymetrix, USA) for the sand fly samples and PureLink PCR Purification Kit (Invitrogen, USA) for the plants. Further, this material was quantified by using fluorometer Qubit^®^ 2.0 (Invitrogen, USA) and purity verified in spectrophotometer NanoDrop™ 2000 (ThermoScientific, USA). Sequencing was carried out in an ABI 3500 automatic sequencer (Applied Biosystems, USA).

Only sequences with a PHRED score above 30 were used in the analysis. Contig assembly was carried out using CodonCode Aligner (CodonCode Corporation). Local alignments were done using BLAST^53^.

### Assembly of DNA barcode library

DNA sequences obtained from each primer were deposited in Genbank database, linked to the National Center for Biotechnology Information (NCBI) (Supplementary Table S1). The deposited sequences were set up for starting a reference library of plant species from the city of Teresina, and from other tropical cities.

### Identification of the plant DNA in sand flies

Each sequence of plant DNA found in the insects was compared to a local plant library using the BLASTN. Only matches with highest score and, at least, 90% identity were considered for the sand flies’ plant feeding preference^54^.

### Statistical Methods

Descriptive statistics were summarized for individual plant characteristics and abiotic factors (*i.e*., temperature, relative humidity and wind speed) across the five trap days. Correlations between the average distance of the plant family from the trap, the average crown expansion of plants from a given family, the proportion of plants in the area from a given family, and the proportion of sand flies testing positive for DNA from a given plant family were investigated for the entire sample of sand flies.

All statistical analyses were performed using R version 3.2.1 (R Foundation for Statistical Computing, Vienna, Austria).

## Additional Information

**How to cite this article**: Lima, L. H. G. M. *et al*. DNA barcode for the identification of the sand fly *Lutzomyia longipalpis* plant feeding preferences in a tropical urban environment. *Sci. Rep.* **6**, 29742; doi: 10.1038/srep29742 (2016).

## Supplementary Material

Supplementary Information

## Figures and Tables

**Table 1:** Abiotic factors and sampling frequency per day of trapping.

Trap day	Temperature (°C)	Air velocity (m/s)	Relative humidity	Number of sand flies caught and analyzed	Total number of plant families detected in the sand fly guts
1	23	0	92	12	7
2	26	1	78	12	6
3	26	0	83	12	5
4	24	1	92	12	8
5	26	0	81	9	4

**Table 2:** Characteristics of plants by family and frequency of sand fly feeding.

Plant Family	Abundance (% of local plant population)	Average Distance from Trap (meters)	Average Crown Expansion (meters)	N (%) Flies with DNA
Anacardiaceae	52 (25.4)	62.3	8.41	2 (3.5)
Bignoniaceae	2 (1.0)	50	9.27	2 (3.5)
Caricaceae	13 (6.3)	72.5	1.68	8 (14.0)
Fabaceae	6 (2.9)	62	8.43	54 (94.7)
Malpighiaceae	17 (8.3)	69.4	3.54	21 (36.8)
Meliaceae	26 (12.7)	62	4.14	1 (1.8)
Myrtaceae	5 (2.4)	60.8	3.97	7 (12.3)
Oxalidaceae	3 (1.5)	60	3.5	22 (38.6)
Rubiaceae	4 (2.0)	73.3	2.52	1 (1.8)
Annonaceae	18 (8.8)	53.4	2.58	0 (0.0)
Musaceae	13 (6.3)	73.1	2.9	0 (0.0)
Rutaceae	25 (12.2)	63.8	3.88	0 (0.0)
Sapindaceae	1 (0.5)	25	12.2	0 (0.0)
Poaceae**	20 (9,8)	14.4	1.09*	0 (0.0)

^*^Average leaf area expansion.

^**^The only species belonging to the Family Poaceae in this study was *Zea mays* (maize), which does not make a crown.
