# Supplementary material for: DNA barcode for the identification of the sand fly Lutzomyia longipalpis plant feeding preferences in a tropical urban environment
Source: Sci Rep. 2016 Jul 20;6:29742. doi: 10.1038/srep29742 (PMC4951712; doi:10.1038/srep29742)
Supplement: Supplementary Information [file srep29742-s1.pdf]

DNA barcode for the identification of the sand fly *Lutzomyia longipalpis* plant feeding preferences in a tropical urban environment

Leonardo H. G. de M. Lima[1,5]*; Marcelo R. Mesquita[2,5]; Laura Skrip[3], Moisés T. de S. Freitas[4]; Vladimir C. Silva[5]; Oscar D. Kirstein[6]; Ibrahim Abassi[6]; Alon Warburg[6]; Valdir de Q. Balbino[4]; Carlos H. N. Costa[1,5]

[1] Federal University of Piauí, Picos, Brazil
[2] Federal Institute of Piauí, Parnaíba, Brazil
[3] Center for Infectious Disease Modeling and Analysis, Yale School of Public Health.
[4] Laboratory of Bioinformatics and Evolutionary Biology, Federal University of Pernambuco, Recife, Brazil
[5] Laboratory of Leishmaniasis, Teresina, Brazil
[6] Department of Microbiology and Molecular Genetics, The Institute of Medical Research Israel-Canada, The Kuvin Centre for the Study of Infectious and Tropical Diseases, The Hebrew University of Jerusalem, Jerusalem, Israel.

* Corresponding author: leonardolima@ufpi.edu.br

Tabela S1: General information on the studied plant species.

Species	Family	Access number herbarium	Access number Genbank	Number of trees (%)	Mean distance to the trap (meters)	Distance from the trap of the closest tree (meters)	Mean crown expansion (meters)
Spondias mombin L.	Anacardiaceae	30.560	KU559208	4 (2.0)	52.0	45	11.43
Mangifera indica	Anacardiaceae	30.728	KU559238	33 (16.1)	60.5	12	7.75
Spondias purpurea L.	Anacardiaceae	30.742	KU559247	3 (1.5)	58.0	47	8.36
Anacardium occidentale	Anarcadiaceae	30.555	KU559209	12 (5.9)	71.9	20	9.20
Annona squamosa	Annonaceae	30.532	KU559199	18 (8.8)	53.4	2	2.58
Tabebuia alba	Bignoniaceae	30.738	KU559229	2 (1.0)	50.0	40	9.27
Carica sp.	Caricaceae	30.732	KU559237	13 (6.3)	72.5	50	1.68
Albizia niopoides Burkat	Fabaceae	30.725	KU559197	2 (1.0)	62.0	56	10.13
Anadenanthera macrocarpa	Fabaceae	30.767	KU559198	1 (0.5)	40.0	40	8.00
Cenostigma macrophyllum	Fabaceae	30.548	KU559211	2 (1.0)	69.5	67	9.18
Tamarindus indica L.	Fabaceae	30.716	KU559249	1 (0.5)	69.0	69	4.00
Malpighia glabra	Malpighiaceae	30.529	KU559194	17 (8.3)	69.4	20	3.54
Azadirachta indica	Meliaceae	30.556	KU559241	26 (12.7)	62.0	24	4.14
Musa sp.	Musaceae	30.737	KU559202	13 (6.3)	73.1	52	2.90
Psidium guajava	Myrtaceae	30.723	KU559228	4 (2.0)	71.5	57	3.58
Syzygium malaccense	Myrtaceae	30.739	KU559231	1 (0.5)	18.0	18	5.55
Averrhoa carambola	Oxalidaceae	30.557	KU559212	3 (1.5)	60.0	35	3.50
Zea mays	Poaceae	30.733	KU559240	20 (9.8)	14.4	7	1.09
Morinda citrifolia L.	Rubiaceae	30.731	KU559242	4 (2.0)	73.3	46	2.52
Citrus sinensis	Rutaceae	30.715	KU559235	14 (6.8)	66.1	19	4.09
Citrus sp	Rutaceae	30.727	KU559236	11 (5.4)	60.8	40	3.61
Talisia esculenta	Sapindaceae	30.719	KU559246	1 (0.5)	25.0	25	12.20
